# Sleep quality and life satisfaction in urban-dwelling solitary Chinese older adults: a study of parallel mediation by depression and cognitive function

**DOI:** 10.3389/fragi.2026.1780401

**Published:** 2026-04-14

**Authors:** Min Wang, Jingli Yu, Qinshan Huang, Shuxun Chi, Luping Qu

**Affiliations:** 1 School of Physical Education, Huzhou University, Huzhou, Zhejiang, China; 2 School of Physical Education, Qufu Normal University, Qufu, Shandong, China; 3 College of Sports and Arts, Jiangxi University of Science and Technology, Ganzhou, Jiangxi, China

**Keywords:** aging population, cross-validation, mediation analysis, mental health, psychological wellbeing, social isolation

## Abstract

**Background:**

With the rapid aging of the global population, the wellbeing of urban-dwelling older adults living alone is linked to increasing research attention.

**Methods:**

Data were derived from the 2017–2018 wave of the Chinese Longitudinal Healthy Longevity Survey (CLHLS). A sample of 1,261 urban-dwelling older adults living alone was analyzed. Sleep quality, depression, cognitive function, and life satisfaction were assessed. Parallel mediation analysis was conducted using the PROCESS macro (Model 4) with 5,000 bootstrap samples. To validate the robustness of the mediation model, a 5-fold cross-validation procedure was performed, ensuring the stability and association consistency of the indirect effects.

**Results:**

Poor sleep quality was significantly associated with higher depression scores and lower cognitive function, both of which were negatively associated with life satisfaction. The mediation analysis revealed that depression and cognitive function independently mediated the relationship between sleep quality and life satisfaction, with depression was associated with a significantly stronger indirect effect (20.22% of total effect) than cognitive function (2.52%). The cross-validated estimates closely matched the full-sample results, confirming model stability.

**Conclusion:**

These findings highlight the crucial role of sleep quality in enhancing life satisfaction among urban solitary older adults, primarily in association with the reduction of depressive symptoms. The study highlights the need for community-based interventions integrating sleep hygiene and depression screening, which is particularly relevant to women living alone.

## 1 Introduction

The global aging population has increased dramatically in the past 2 decades, with the proportion of individuals aged 65 and above rising by 27% according to the World Health Organization’s statistics (Xie et al., 2020). This trend is particularly pronounced in urban areas, where solitary living among older adults has become increasingly prevalent. Urban solitary older adults face a host of unique challenges, including social isolation, cognitive decline, and mental health issues such as depression, all of which are negatively associated with their life satisfaction (Simões et al., 2020). Cognitive decline, characterized by memory loss, attention deficits, and slowed thinking, not only is associated with impaired daily functioning, but also is linked to safety risks and correlated with increased vulnerability to anxiety and depression, further lowering life satisfaction (Adcock et al., 2020).

Relevant government data showed that by the end of 2021, the older adults aged 60 and above in China had reached 267 million (Ministry of Civil Affairs of the People’s Republic of China, https://www.mca.gov.cn/), and according to “Basic Data Bulletin of the Fifth Sampling Survey on the Living Conditions of Urban and Rural Elderly People in China”, older adults living alone in urban areas account for 12.4% (China National Committee on Aging, https://www.cncaprc.gov.cn/). Unlike their non-solitary peers, this group confronts a dual predicament: on one hand, the lack of daily companionship and immediate care elevates the risk of unaddressed health issues, such as unmonitored chronic disease progression; on the other hand, the absence of regular social interaction amplifies the fragility of their mental and cognitive health (Salvador, 2023). While prior studies have explored the link between sleep and life satisfaction in the general older adults, few have focused on the urban solitary subgroup. In this population, the interplay of sleep disorders, depression, and cognitive decline may be more prominent due to the absence of social buffering effects, creating a critical research gap in targeted geriatric care that this study seeks to address.

Life satisfaction is a key measure of quality of life, especially for urban, solitary elderly individuals, and is closely connected to both physical and mental health (Kim et al., 2021). Sleep, a cornerstone of physiological and psychological wellbeing, profoundly influences life satisfaction. Physiologically, sleep is essential for bodily repair, cell regeneration, hormone secretion, and immune system enhancement. Adequate sleep is associated with better physical function, higher immunity, and lower risk of disease (Baranwal et al., 2023). Psychologically, sleep plays a key role in emotional regulation, which is associated with better mental health. A study has established a strong correlation between sleep disturbances and depressive symptoms among urban older adults (Yang et al., 2021), and also confirmed that sleep supports memory, attention, and brain health more broadly (Mason et al., 2021). Prior work has also noted that better sleep quality and sufficient sleep duration correlate with higher life satisfaction (Varghese et al., 2020), though this relationship may be moderated by factors such as psychological state, lifestyle, and social support (Baranwal et al., 2023).

Depression and cognitive function are key intermediaries linking sleep quality and life satisfaction in this population. Depression is particularly prevalent among urban solitary older adults, often correlated with loneliness and lack of social support (Isik et al., 2021), and there is a well-documented bidirectional relationship between sleep problems and depression—sleep disorders are closely associated with depression, while depressive symptoms are also linked to poorer sleep disturbances, creating a vicious cycle that is negatively associated with life satisfaction. Cognitive decline is another pressing issue for urban solitary older adults: poor sleep quality or insufficient sleep is associated with accelerated cognitive deterioration, which not only is linked to reduced independence but also is correlated with increased psychological distress like depression and anxiety, compounding the challenges this group faces (Robbins et al., 2020).

Notably, while sleep is recognized as a critical factor associated with elderly wellbeing (Yuliati et al., 2025), adequate sleep is associated with better emotional regulation and cognitive maintenance (Casavi et al., 2022), deficient sleep is associated with depressive symptoms and cognitive impairment (Vanek et al., 2020), existing research has two critical limitations. First, most studies overlook the specific context of urban solitary older adults, whose lack of social support may amplify the negative impacts of poor sleep. Second, few have explored the parallel mediating roles of depression and cognitive function in the relationship between sleep quality and life satisfaction. The urban solitary context further strengthens these variable interconnections: unaddressed sleep disturbances due to lack of family companionship raise depression risk (Wakefield et al., 2020), and without daily social interaction to stimulate cognition, sleep-induced cognitive decline is more likely to progress to functional impairment and reduced life satisfaction (Yu et al., 2025). The mechanism emphasizes the necessity for targeted research on this population.

To address these research gaps, this study is grounded in an integrated framework that combines the Stress Process Model (Pearlin et al., 1981) and the Reserve Capacity Model (Gallo and Matthews, 2003). The Stress Process Model posits that chronic stressors deplete psychological resources, such as poor sleep, leading to adverse mental health outcomes. In the context of urban solitary older adults, poor sleep is conceptualized as a persistent stressor that may erode emotional and cognitive reserves. The Reserve Capacity Model further suggests that, due to factors such as social isolation, individuals with diminished reserves are more vulnerable to negative health outcomes. Together, these models provide a rationale for examining depression and cognitive function as parallel mediators linking sleep quality to life satisfaction, particularly in a population lacking social buffers. On this basis, we propose that higher sleep quality is positively associated with life satisfaction among urban solitary older adults, given that sufficient and high-quality sleep helps maintain physical and mental balance and thus improves overall life evaluations; meanwhile, depression is expected to mediate the sleep quality-life satisfaction relationship because poor sleep disrupts serotonin secretion, which induces depressive emotions and further reduces life satisfaction, and cognitive function is also hypothesized to act as a mediator in this relationship as sleep deprivation can cause hippocampal atrophy, impair cognitive functioning, and ultimately lower life satisfaction.

## 2 Methods

### 2.1 Participants

Data for this study were derived from the Chinese Longitudinal Healthy Longevity Survey (CLHLS) of Peking University. This survey conducted eight rounds of visits in randomly sampled counties and cities in 23 provinces, municipalities, and autonomous regions across the country from 1998 to 2018, covering approximately 1.103 million households.

The data of this study were derived from the 2017–2018 CLHLS, with a total of 15,874 samples, which is a nationally representative longitudinal survey with strict sampling and quality control procedures (Pei et al., 2022). Although the data is 7 years old by the end of 2025, the core characteristics of the older adults’ sleep and mental health (e.g., the prevalence of sleep disorders, the mediating role of depression) have shown high stability in China over the past decade (Yao et al., 2025). Moreover, the CLHLS dataset remains the most comprehensive public data source for Chinese older adults’ health research, and subsequent studies can verify this findings using the latest 2022–2023 CLHLS wave data.

To focus on the living conditions of elderly individuals living alone in urban areas, the sample was strictly screened to exclude (Xie et al., 2020): Non-solitary individuals. According to the survey definition of CLHLS, living alone was specifically defined as the elderly person living alone in daily life without other family members or non-family members living together for a long time. The data on the living alone status was obtained through the question “Who do you currently live with?” in the questionnaire. The options included “living alone”, “living with a spouse”, and “living with children or other family members”. Only the respondents who chose “living alone” were retained in this study, and those who lived with a spouse, children, or other family members were excluded. The reason for selecting the solitary samples was that older adults living alone may face greater challenges in mental health and cognitive function due to the lack of a daily social support system, which was closely related to the goal of this study. Excluding non-solitary samples made the research results more targeted and reliable (Simões et al., 2020). Participants residing in rural areas. Based on the residence information of the respondents, all samples of older adults living in rural areas were also excluded. The exclusion of rural samples was based on significant urban-rural disparities in three key dimensions (Xie et al., 2020): Medical accessibility: Urban older adults have better access to sleep disorder diagnosis and mental health services, while rural older adults face greater barriers (Muench et al., 2022), which would confound the relationship between sleep quality and mental health (Simões et al., 2020); Social support structure: Rural solitary older adults often rely on village collective care, while urban counterparts lack such support (Haohua, 2024), leading to different mediating mechanisms (Adcock et al., 2020); Lifestyle patterns: Urban older adults have more sedentary lifestyles, while rural older adults retain more physical labor, which affects sleep duration and cognitive status (Wu et al., 2024). Controlling for these urban-rural differences ensures that the results accurately reflect the characteristics of urban solitary older adults, avoiding the dilution of core findings by heterogeneous samples.

After applying these criteria, 1,261 participants were retained for analysis. This sample group was more representative and could better reflect the living status of older adults living alone in cities. This study has been approved by the Ethics Review Committee of Peking University (approval number: IRB00001052-13074), and all participants signed written informed consent forms. The CLHLS dataset is an open database and is freely available to the academic community and the public through the Peking University Open Research Data Platform (http://opendata.pku.edu.cn).

### 2.2 Data collection

#### 2.2.1 Social demographic variables

The survey collected the basic social demographic characteristics of the respondents, such as age, gender, marital status, and ethnicity; physical condition indicators included weight, height, waist circumference, hip circumference, and heart rate; lifestyle and health behaviors covered smoking, drinking, exercise, physical activity, housework, as well as hearing, vision, and chronic diseases. In addition, the socioeconomic status of the respondents (such as years of education and sources of income), sleep duration and quality, depressive symptoms, cognitive function, and life satisfaction were also investigated.

#### 2.2.2 Sleep status survey

In this study, the sleep status of the respondents was evaluated from two dimensions: sleep quality and sleep duration. Specifically, the sleep duration was quantified and recorded through the question “How many hours do you usually sleep a day?” in the questionnaire, and the sleep quality was subjectively evaluated through the question “How is your current sleep quality?” Respondents could choose the option that best matched their actual situation from “excellent”, “good”, “average”, “poor”, or “very bad”. Responses were coded as follows: 1 = excellent, 2 = good, 3 = average, 4 = poor, 5 = very bad (Lin et al., 2022; ZHANG et al., 2024).

#### 2.2.3 Depression status evaluation

The Center for Epidemiologic Studies Depression Scale - 10 (CES - D10) was selected to assess the level of depression in this study. This scale has been widely used in the field of geriatric mental health research due to its comprehensive coverage of depressive symptoms and good reliability and validity in older adults. The Cronbach’s alpha coefficient of this questionnaire in this study was 0.928, which is in line with the high reliability standard reported in previous studies (Yang et al., 2024). Additionally, compared with other similar scales, CES - D10 has the advantage of being relatively concise and easy to administer, making it suitable for large-scale surveys of older adults. The scale listed 9 items about the behavior and emotional problems of the respondents in the past week. The response frequency was scored from 1 to 5, and the total score ranged from 9 to 45. The higher the score, the greater the risk of depression and the poorer the mental health status.

#### 2.2.4 Cognition assessment

The Mini-Mental State Examination (MMSE) was used to measure cognitive function. This scale was developed by Folstein et al., in 1975 and evaluated multiple aspects of abilities such as orientation, attention, memory, language, and naming (Gao et al., 2021). CLHLS adjusted some items according to China’s national conditions. For example, subtracting 7 from 100 was changed to subtracting 3 from 20 continuously. The full score of this scale was 30, and the higher the score, the better the cognitive function. The Cronbach’s alpha coefficient of this scale in this study was 0.872 (Xing et al., 2024). According to previous studies, a score below 24 was considered cognitive impairment, and a score of 24 and above was considered normal cognitive function.

#### 2.2.5 Life satisfaction assessment

Life satisfaction was assessed through three questions in the questionnaire: “How do you feel about your current life?” “How do you feel about your current health status?” and “Has your health status changed in the past year?” Respondents could choose “very good”, “good”, “average”, “bad”, or “very bad” as their answers to evaluate their life satisfaction status (Zhang et al., 2022).

It should be noted that the study adopted a cross-sectional design based on CLHLS 2017–2018 data, which can only reveal the correlation between variables rather than infer causal relationships. For example, while this study observed the association between poor sleep quality and high depression scores, it cannot determine whether sleep disturbance precedes depressive symptoms or *vice versa*. To address this, this study controlled for key confounding variables in the statistical model to reduce the interference of extraneous factors on the core associations, such as including chronic disease status, years of education, and income source.

### 2.3 Statistical analysis

Data were analyzed using SPSS 26.0 and the PROCESS plug-in (version 4.1) for mediation analysis. Descriptive statistics and Spearman correlations were computed. The Bootstrap method (5,000 resamples, 95% confidence interval) was applied to test mediation effects. To maintain data integrity, all 1,261 samples that met the screening criteria were included. The Kolmogorov - Smirnov test was employed to assess the normality of the data, and a p - value greater than 0.05 indicated that the data approximately followed a normal distribution. Initially, the independent sample t - test and χ^2^ test were utilized to examine the homogeneity of each variable. Subsequently, the Spearman correlation analysis method was applied to evaluate the correlation among sleep, depression, cognitive function, and life satisfaction. The Process plug - in 4.1 was used to construct the mediation model. This plugin is specifically designed for analyzing mediation effects and provides a convenient and efficient way to test the significance of indirect effects through methods such as Bootstrap sampling. In this study, Bootstrap sampling was conducted 5,000 times with a 95% confidence interval to ensure the robustness of the results. The range of the correlation coefficient was as follows: 0 to 0.19 indicated no correlation, 0.20 to 0.39 was a low correlation, 0.40 to 0.59 was a moderate correlation, 0.60 to 0.79 was a relatively high correlation, and ≥0.80 was a high correlation (Song and Park, 2020). In addition, the Process plug-in 4.1 was used to build a model, and the Bootstrap method was used to test the significance of the mediating effect. Bootstrap sampling was performed 5,000 times, and a 95% confidence interval was set. A P < 0.05 was considered statistically significant.

### 2.4 Internal validation: cross-validation procedure

To assess the robustness of the mediation model and mitigate potential overfitting, the study performed an internal validation using 5-fold cross-validation. The total sample (N = 1,261) was first randomly partitioned into five equally sized subgroups (folds). In each of the five iterations, one fold was held out as the test set, while the remaining four folds were combined to form the training set. The parallel mediation model (PROCESS Model 4) with all specified covariates was fitted on the training set to obtain path coefficients. These coefficients were then applied to the test set to calculate the indirect effects. This process was repeated five times so that each fold served as the test set once. The final cross-validated indirect effect estimates were computed as the mean of the effects obtained from the five test sets, providing an evaluation of the model’s stability and generalizability within the available sample.

## 3 Results

### 3.1 Descriptive statistics and preliminary analyses

Demographic and clinical characteristics of the 1,261 participants, stratified by gender, are summarized in Table 1. The sample included 476 men (37.8%) and 785 women (62.2%), with slight variations in group sample sizes due to missing values for specific variables. Women reported significantly poorer sleep quality, higher levels of depressive symptoms, and lower cognitive function compared to men (all p < 0.01). No gender difference was observed in life satisfaction (p = 0.812).

**TABLE 1:** Demographic and health characteristics of the study sample.

Variables	Men (mean ± SD)	Women (mean ± SD)	p	Cohen’s d
Age (years)	85.37 ± 10.03	85.32 ± 9.81	0.936	0.005
Weight (kg)	59.33 ± 13.91	51.95 ± 25.15	<0.001	0.362
Height (cm)	161.85 ± 9.79	149.81 ± 10.51	<0.001	1.168
Years of education (years)	4.80 ± 4.60	2.42 ± 3.98	<0.001	0.523
Sources of income	3.71 ± 2.48	3.36 ± 2.25	0.013	0.143
Smoking (1: yes; 2: no)	1.85 ± 1.14	2.54 ± 1.95	<0.001	−0.438
Drinking (1: yes; 2: no)	2.10 ± 1.44	2.55 ± 1.95	<0.001	−0.261
Exercising (1: yes; 2: no)	1.56 ± 0.50	1.68 ± 0.47	<0.001	−0.250
Taking nutritional supplements (1: yes; 2: no)	1.90 ± 0.31	1.87 ± 0.34	0.123	0.089
Doing physical labor (1: yes; 2: no)	1.27 ± 0.44	1.33 ± 0.47	0.034	−0.121
Doing housework	2.41 ± 1.83	2.13 ± 1.75	0.007	0.158
Hearing normal or not (1: yes; 2: no)	1.39 ± 0.84	1.39 ± 0.88	0.999	0.000
Vision status	1.42 ± 0.73	1.52 ± 0.79	0.013	−0.131
Chronic diseases or not	2.46 ± 0.75	2.56 ± 0.72	0.016	−0.138
Sleep quality	2.58 ± 1.25	2.85 ± 1.34	<0.001	−0.203
Sleep duration (hours)	7.46 ± 2.43	6.87 ± 2.18	<0.001	0.246
Depression (score)	21.18 ± 4.55	22.21 ± 4.38	<0.001	−0.234
Cognitive function (score)	26.48 ± 7.65	25.22 ± 8.00	0.005	0.159
Life satisfaction (score)	7.46 ± 1.85	7.48 ± 1.91	0.812	−0.015

1 Data are presented as Mean ± Standard Deviation. The total sample size was N = 1,261; group sample sizes varied slightly due to missing values in specific variables.

^2^Variablecoding. Sources of income: 1 = pension; 2 = spouse; 3 = children; 4 = grandchildren; 5 = other relatives; 6 = local government or community; 7 = self-labor or work; 8 = others; Doing housework: 1 = almost daily; 2 = not daily but at least once a week; 3 = not weekly but at least once a month; 4 = not monthly but sometimes; 5 = not participating; Vision status: 1 = able to see and distinguish notch direction; 2 = able to see but cannot distinguish notch direction; 3 = cannot see clearly; 4 = blind; Chronic diseases: 1 = normal; 2 = single chronic disease; 3 = multiple chronic comorbidities; Sleep quality: 1 = very good; 2 = good; 3 = average; 4 = bad; 5 = very bad; 8 = unable to answer; Life satisfaction: Scores range from 3 to 15, with higher scores indicating worse satisfaction.

^3^Effect sze categories (Cohen’s d). Trivial: |d| < 0.2; Small: 0.2 ≤|d|< 0.5; Medium: 0.5 ≤|d|< 0.8; Large: |d|≥ 0.8.

Bivariate correlations (Table 2) revealed that poorer sleep quality was significantly associated with more severe depression (r = 0.254, p < 0.001), lower cognitive function (r = −0.403, p < 0.001), and worse life satisfaction (r = 0.273, p < 0.001). These correlations satisfied the preliminary conditions for mediation analysis.

**TABLE 2:** Bivariate correlations among key study variables.

Variables	1	2	3	4	5
1. Sleep quality	1	−0.410^**^	0.254^**^	−0.403^**^	0.273^**^
2. Sleep duration	−0.410^**^	1	−0.134^**^	−0.077^**^	−0.144^**^
3. Depression	0.254^**^	−0.134^**^	1	−0.067*	0.293^**^
4. Cognitive function	−0.403^**^	−0.077^**^	−0.067^*^	1	−0.138^**^
5. Life satisfaction	0.273^**^	−0.144^**^	0.293^**^	−0.138^**^	1
Mean	2.749	7.090	21.813	25.699	7.472
SD	1.312	2.297	4.4730	7.892	1.889

** indicates significance at the 0.01 level (two-tailed); * indicates significance at the 0.05 level (two-tailed).

According to Baron and Kenny’s approach (Baron and Kenny, 1986), the prerequisite for verifying the independent mediating effects of depressive symptoms and cognitive function, as well as the parallel mediating effect of sleep quality on the life satisfaction of urban solitary older adults, is that there are significant correlations among the four variables. If the association between them becomes 0 after adjusting for the mediators, it belongs to a complete mediating variable; if the association weakens after adjusting for the mediators, it belongs to a partial mediating variable. The results of the correlation analysis in the previous stage showed that there were close relationships among the four variables, which supported proceeding with the mediation analysis.

### 3.2 Parallel mediation model analysis

We tested a parallel mediation model wherein sleep quality influenced life satisfaction through the independent pathways of depression and cognitive function. Path coefficients, derived from bootstrap analysis (5,000 samples), are presented in Table 3. Sleep quality was significantly positively associated with depression (β = 1.183, p < 0.001) and significantly negatively associated with cognitive function (β = −0.378, p = 0.020). Both depression (β = 0.095, p < 0.001) and cognitive function (β = −0.036, p < 0.001) were significantly associated with life satisfaction. The direct effect of sleep quality on life satisfaction remained significant after accounting for the mediators (β = 0.428, p < 0.001).

**TABLE 3:** Path coefficients for the parallel mediation model.

Path	β	SE	t	p	Bootstrap 95% CI
X→M1	1.183	0.138	8.567	<0.001	[0.912, 1.454]
X→M2	−0.378	0.168	−2.320	0.020	[-0.697, −0.058]
M1→Y	0.095	0.013	7.525	<0 0.001	[0.070, 0.120]
M2→Y	−0.036	0.011	−3.392	<0 0.001	[-0.057, −0.015]
X→Y (direct effect)	0.428	0.057	7.472	<0.001	[0.315, 0.540]

1. X, sleep quality; M1, depression; M2, cognitive function; Y, Life Satisfaction. Unstandardized coefficients (β) are reported. All models controlled for age, education, and chronic disease status; 2. Relative difference = |(CV, Mean − Full Sample Value)/Full Sample Value| × 100%.

The bootstrapping results for indirect effects are detailed in Table 4. The total indirect effect was significant (effect = 0.126, 95% CI [0.082, 0.175]), accounting for 22.74% of the total effect, indicating that 22.74% of sleep quality on life satisfaction is transmitted through depression and cognitive function. The indirect effect through depression was significant and substantial (effect = 0.112, 95% CI [0.072, 0.158]; 20.22% of total effect), meaning poorer sleep quality is associated with higher depression levels, which in turn are linked to worse life satisfaction—this is a small but meaningful effect in community-dwelling older adults. In contrast, the indirect effect through cognitive function, while significant, was markedly smaller (effect = 0.014, 95% CI [0.002, 0.030]; 2.52% of total effect), suggesting sleep quality’s influence on life satisfaction via cognitive function is minimal. A formal contrast confirmed that the mediation via depression was significantly stronger than via cognitive function (difference = 0.098, 95% CI [0.056, 0.147]).

**TABLE 4:** Direct, indirect, and total effects of the parallel mediation model.

Effect type	Effect size	Boot SE	Bootstrap 95% CI	Relative mediating effect
Total effect	0.554	0.057	[0.442, 0.666]	100%
Direct effect	0.428	0.057	[0.315, 0.540]	77.26%
Total indirect effect	0.126	0.023	[0.082, 0.175]	22.74%
- M1	0.112	0.022	[0.072, 0.158]	20.22%
- M2	0.014	0.007	[0.002, 0.030]	2.52%
(C1)	0.098	0.023	[0.056, 0.147]	-

1. X, sleep quality; M1, depression; M2, cognitive function; Y, life satisfaction; 2. C1 = Indirect effect via M1 − Indirect effect via M2. Boot SE, bootstrap standard error. 3. Relative Mediating Effect = (Effect Size/Total Effect) × 100%. Bootstrap 95% confidence intervals (CI) are based on 5,000 samples.

### 3.3 Internal validation via 5-fold cross-validation

To evaluate the robustness of the parallel mediation model, an internal validation was performed using 5-fold cross-validation (5-CV). The core procedure was as follows: all samples were randomly and equally divided into 5 subsets. In each iteration, 4 subsets were used as the training set to fit the model, and the remaining 1 subset was used as the test set to verify predictive performance. This process was repeated 5 times to ensure each subset served as the test set exactly once. All data preprocessing steps, including missing value removal and Z-score standardization, were completed before sample splitting to avoid information leakage. Results showed high stability of path coefficients across folds (Table 5), with mean cross-validated estimates nearly identical to full-sample coefficients (all relative differences <3%).

**TABLE 5:** Stability of path coefficients and effect sizes via 5-fold cross-validation.

Variables	Fold 1	Fold 2	Fold 3	Fold 4	Fold 5	CV (Mean ± SD)	Full sample	Relative difference
Path								
X→M1	1.168	1.195	1.172	1.202	1.176	1.183 ± 0.015	1.183	0.00%
X→M2	−0.381	−0.373	−0.376	−0.385	−0.379	−0.379 ± 0.005	−0.378	0.26%
M1→Y	0.093	0.097	0.094	0.097	0.093	0.095 ± 0.002	0.095	0.00%
M2→Y	−0.037	−0.036	−0.034	−0.039	−0.039	−0.037 ± 0.002	−0.036	2.78%
Effect type								
Test set R^2^	0.124	0.132	0.145	0.131	0.129	0.132 ± 0.008	0.145	8.97%
Total effect	0.556	0.550	0.563	0.551	0.554	0.555 ± 0.005	0.554	0.18%
Direct effect	0.433	0.422	0.440	0.419	0.429	0.429 ± 0.009	0.428	0.23%
- M1	0.109	0.116	0.110	0.117	0.110	0.112 ± 0.003	0.112	0.00%
- M2	0.014	0.013	0.013	0.015	0.015	0.014 ± 0.001	0.014	0.00%

X, sleep quality; M1, depression; M2, cognitive function; Y, Life Satisfaction. CV, coefficient of variation; SD, standard deviation; 2. Relative difference = |(CV, Mean − Full Sample Value)/Full Sample Value| × 100%.

Similarly, the test set mean R^2^ was 0.132 (SD = 0.008), with a relative difference of only 8.97% compared to the full-sample model *R* ^2^ (0.145). For psychological and epidemiological models, an *R* ^2^ difference <10% across sample subsets is considered clinically acceptable, indicating the model’s predictive ability is stable. Additionally, key effect size estimates (total, direct, and indirect effects) demonstrated excellent stability (Table 5), with cross-validated means differing from full-sample estimates by less than 0.25%, such as total effect: 0.555 vs. 0.554; direct effect: 0.429 vs. 0.428. These results confirm the reliability and generalizability of the primary findings to similar older adult populations.

## 4 Discussion and analysis

This study systematically explored the impact of sleep quality on life satisfaction among elderly individuals living alone in urban areas, examined the parallel mediating roles of depression and cognitive function, and analyzed the gender differences. To address concerns regarding model overfitting, it employed a rigorous internal cross-validation strategy. The close agreement between the cross-validated effect estimates and those from the full-sample model underscores the internal robustness and reproducibility of the identified mediation pathways, particularly the dominant role of depression.

Female reported poorer sleep quality, higher depression scores, and lower cognitive function than male, while no significant gender difference was found in life satisfaction from Table 1. This result is consistent with previous studies showing that women’s sleep disturbances are correlated with hormonal fluctuations (Zhou et al., 2021), especially postmenopausal women whose estrogen decline is associated with disrupted sleep-wake cycles and higher depression risk. Men’s higher education levels and more diverse income sources may be associated with reduced negative effects of sleep problems on cognitive function, which explains their better cognitive performance. The absence of gender difference in life satisfaction may be due to the same living constraints faced by urban solitary older adults, such as loneliness and health concerns, which offset the gender-specific differences in sleep and mental health (Takagi et al., 2020). No gender difference in life satisfaction was also reported in a cross-regional study (Joshanloo and Jovanović, 2020), which supports the robustness of this finding.

Furthermore, sleep quality emerged as a pivotal predictor of life satisfaction among urban solitary older adults, exerting both direct effects and indirect influences via depression and cognitive function. Psychologically, poor sleep is associated with physical and mental exhaustion, which is linked to depressive emotions and a negative life that is directly associated with lower life satisfaction (Perotta et al., 2021). Neurobiologically, insufficient sleep is associated with alterations in key neurotransmitter systems and brain structure changes, and accelerates cognitive decline, all of which are correlated with higher depression risk and poorer daily functioning (Bishir et al., 2020; Bajaj, 2020). For urban solitary older adults, who already face social isolation and limited support, poor sleep is associated with stronger feelings of loneliness and reduced social participation, while cognitive decline is also linked to impaired communication and reduced independence, creating a cascading pattern associated with lower life satisfaction (Papi and Cheraghi, 2021; Khodabakhsh, 2022).

The finding of parallel mediation underscores that sleep quality influences life satisfaction through two distinct, yet simultaneous, pathways. The significantly stronger indirect effect via depression suggests that the emotional pathway is primary, while the cognitive pathway, though significant, plays a more subsidiary role. This pattern aligns with theories positing that affective states are more proximal determinants of global life evaluations than cognitive abilities. Mediation analyses revealed that both depression and cognitive function were significantly associated with life satisfaction, with depression exerting a stronger indirect effect in Table 4. This notable difference in the mediating effect size (20.22% for depression vs. 2.52% for cognitive function) can be comprehensively explained by three interrelated factors, combining psychological mechanism, adaptive buffering, and cultural context characteristics of Chinese urban solitary older adults. First, emotional states are more proximal and direct in their association with global life satisfaction than cognitive abilities (Tamir, 2021), depressive feelings are closely linked to an overall negative evaluation of life, while cognitive function decline is only associated with impairments in specific daily task management and not consistently linked to reduced life satisfaction (Kim et al., 2021). Second, the potential negative association of cognitive decline with life satisfaction is effectively buffered by adaptive behaviors and social support in this population (Wang et al., 2021; Gaia et al., 2021), urban solitary older adults can adjust their lifestyles to adapt to mild cognitive decline, and even limited community or social support can offset the impact of cognitive impairment on life perception. Third, the cultural context of collectivism in China further amplifies the mediating effect of depression, emotional distress is often less openly addressed among Chinese older adults, especially those living alone who lack familial emotional communication, leading to unmitigated depressive symptoms that are more strongly associated with life satisfaction; in contrast, cognitive decline is often perceived as a “normal aging phenomenon” in Chinese society, and family/community care can partially compensate for cognitive impairments, thus weakening the mediating link between cognitive function and life satisfaction. The stronger mediating effect of depression may be understood within the Chinese cultural context, where emotional wellbeing is closely tied to familial harmony and social roles. For urban solitary older adults, the lack of daily familial interaction may exacerbate feelings of loneliness and uselessness, amplifying the impact of sleep-related emotional distress on life satisfaction. Additionally, in collectivist societies, cognitive decline may be partially offset by familial support or community care, whereas emotional distress, which is often stigmatized and less openly addressed, may remain unmitigated. This aligns with studies suggesting that in Asian cultures, emotional health is a stronger predictor of life satisfaction than cognitive function, particularly among those living alone (Khodabakhsh, 2022).

Urban solitary older adults can mitigate the effects of cognitive impairment through lifestyle adjustments, and robust social support enables maintenance of high life satisfaction despite cognitive decline. For example, Wang et al. noted that elderly individuals could adapt to cognitive decline by altering their lifestyles (Wang et al., 2021), while Gaia et al. found that elderly individuals with robust social support could sustain high life satisfaction despite cognitive decline (Gaia et al., 2021). In contrast, emotional health has a more direct and unbuffered impact on life satisfaction, as demonstrated by a prior study (Kim et al., 2021) showing emotional wellbeing is a stronger predictor of life quality than cognitive function. This findings are also consistent with existing studies on the mediating roles of depression and cognitive function in the sleep-life satisfaction relationship (Wang et al., 2023; Banerjee and Boro, 2022), but the relative strength of the mediating effects differs from some previous reports. For example, a prior Indian study (Banerjee and Boro, 2022) reported a stronger cognitive mediation, a difference that may stem from cultural differences in the valuation of cognitive independence. In societies with stronger collective family support, cognitive decline might pose a greater threat to one’s perceived social role and thus life satisfaction.

Based on the core findings, we propose targeted community-based intervention solutions for urban solitary older adults. First, sleep hygiene intervention. Launch free sleep health workshops in urban communities to provide personalized sleep guidance for solitary older adults, including regular sleep schedule formulation, sleep environment optimization and non-pharmacological sleep improvement methods like gentle meditation and breathing exercises. Establish a sleep quality screening system to enable early identification of solitary older adults with poor sleep quality. Second, depression screening and intervention. Integrate brief depression screening with CES-D10 into routine community health checks for urban solitary older adults and set up free psychological counseling points in communities to offer emotional support for those with high depressive symptoms. For solitary elderly women, the high-risk group in this study, organize gender-specific peer support groups and alleviate depressive emotions and loneliness through regular social interaction. Third, cognitive function protection. Design low-threshold cognitive stimulation activities for urban solitary older adults, such as calligraphy, chess and memory training classes, and combine cognitive training with mild physical exercise like tai chi and walking to enhance the association between better cognitive function and sleep quality. Conduct regular cognitive function assessments to monitor changes in the cognitive status of this population. In addition, integrate the three intervention dimensions into a unified community health service system, conduct regular follow-ups and make personalized adjustments to intervention plans to ensure the effectiveness of the intervention measures.

This study provides critical empirical evidence for improving the wellbeing of urban solitary older adults, while internal cross-validation supports the model’s robustness within sample, the primary limitation remains the lack of validation in a fully independent, external cohort. Future research should aim to replicate these findings in separate populations of urban-dwelling older adults living alone to confirm generalizability. Furthermore, this study has several limitations. First, the cross-sectional design limits causal inference. For example, the observed associations may reflect the bidirectional relationship between sleep disturbance and depression; that is, sleep problems may trigger depression, or depressive symptoms may worsen sleep. Additionally, unmeasured confounders such as the intensity of social support and access to daily care services may have confounded the results, and these factors should be explicitly incorporated into subsequent investigations. Second, the sample only covers 23 provinces, limiting the generalizability of findings to the broader Chinese urban solitary older adults. Future research should focus on three core directions: expanding the sample size to bolster the external validity of study outcomes; adopting longitudinal research designs to dynamically monitor long-term fluctuations in sleep quality, depression severity, cognitive function, and life satisfaction, thereby clarifying the causal pathways among these variables; exploring additional mediating or moderating factors, such as social support and economic status, to construct more holistic and targeted intervention frameworks for this vulnerable group.

## 5 Conclusion

This study highlights the significant impact of sleep quality on life satisfaction, primarily associated with depression and secondarily correlated with cognitive function. Given the dominant mediating role of depression, community-based interventions for this population should prioritize integrating brief depression screening into routine health checks, which is associated with sleep hygiene workshops. For elderly women living alone, tailored social activities involving mild exercise and peer support are particularly relevant to this group. These findings are associated with a theoretical basis for future research aimed at improving the wellbeing of this vulnerable population.

## Data Availability

The datasets presented in this study can be found in online repositories. The names of the repository/repositories and accession number(s) can be found in the article/Supplementary Material.
